# Plaster of Paris and How to Use It

**Published:** 1907-03

**Authors:** 


**Plaster of Paris and How to Use It.** By **Martin W. Ware.,** M.D., Adjunct Attending Surgeon, Mount Sinai Hospital; Surgeon to the Good Samaritan Dispensary; Instructor in Surgery, N. Y. Post-Graduate Medical School. 12mo; 72 illustrations, about 100 pages. New York: Surgery Publishing Co. (Cloth, $1.00).

Occasionally out of the raft of books on a variety of medical subjects there comes a little volume on a commonplace topic which, by very reason of its familiarity, has been neglected. One such is Plaster of Paris and How to Use It, and it takes its place among the really valuable books in a medical library by pure right of conquest. There is a demand for such instruction as is contained in this book. If there isn't there ought to be when one considers the mud-pie methods so frequently seen in surgical practice. This isn't a hardened, corrugated, superior sort of a book. On the contrary it is a narrative, with a prodigality of illustrations which tells all about the uses to which plaster of paris may be put in surgery, from the making of a bandage to its use as splints and corsets. It will be more or less of a jolt to many users of plaster to find out how little they really know about the manner of handling it, after they have read this book. It is worth while. In spite of the fact that it makes one aware of his shortcomings it doesn't bite into one's vanity very viciously.

